# Translational Research on Azacitidine Post-Remission Therapy of Acute Myeloid Leukemia in Elderly Patients (QOL-ONE Trans-2)

**DOI:** 10.3390/ijms252111646

**Published:** 2024-10-30

**Authors:** Esther Natalie Oliva, Maria Cuzzola, Matteo Della Porta, Anna Candoni, Prassede Salutari, Giuseppe A. Palumbo, Gianluigi Reda, Giuseppe Iannì, Matteo Zampini, Saverio D’Amico, Giovanni Tripepi, Debora Capelli, Caterina Alati, Maria Concetta Cannatà, Pasquale Niscola, Bianca Serio, Santina Barillà, Pellegrino Musto, Ernesto Vigna, Lorella Maria Antonia Melillo, Rocco Tripepi, Maria Elena Zannier, Yasuhito Nannya, Seishi Ogawa, Corrado Mammì

**Affiliations:** 1 Hematology Unit, Grande Ospedale Metropolitano Bianchi Melacrino Morelli, 89124 Reggio di Calabria, Italy; caterina.alati@gmail.com (C.A.); santina.barilla@gmail.com (S.B.); 2 UOSD Tipizzazione Tissutale, Grande Ospedale Metropolitano Bianchi Melacrino Morelli, 89124 Reggio di Calabria, Italy; mariellacuzzola@gmail.com; 3 IRCCS Humanitas Research Hospital, 20089 Milan, Italy; matteo.della_porta@hunimed.eu (M.D.P.); matteo.zampini@humanitasresearch.it (M.Z.); saverio.damico@humanitas.it (S.D.); 4 Clinica Ematologica, ASUFC, University of Udine, 33100 Udine, Italy; annacandoni@gmail.com (A.C.); zanniermariaelena@gmail.com (M.E.Z.); 5 Dipartimento Oncologico-Ematologico Ospedale Civile Spirito Santo Pescara, 65124 Pescara, Italy; prassede.salutari@gmail.com; 6 Dipartimento di Scienze Mediche Chirurgiche e Tecnologie Avanzate “G.F. Ingrassia”, University of Catania, 95123 Catania, Italy; palumbo.gam@gmail.com; 7 Hematology Department, Foundation IRCCS Ca’ Granda, Ospedale Maggiore Policlinico, University of Milan, 20100 Milan, Italy; gianluigi.reda@policlinico.mi.it; 8 Dielnet SRL, CRO Reggio Calabria, 89124 Reggio Calabria, Italy; ianni@dielnet.it; 9 IFC-CNR Institute of Clinical Physiology Reggio Calabria, 89124 Reggio Calabria, Italy; gtripepi@hotmail.com; 10 Clinica di Ematologia Azienda Ospedaliera Universitaria, Ospedali Riuniti di Ancona, 60126 Ancona, Italy; d.capelli@ospedaliriuniti.marche.it; 11 UOSD Medical Genetics, Great Metropolitan Hospital, 89124 Reggio Calabria, Italy; mccannata@gmail.com (M.C.C.); corradomammi@tiscali.it (C.M.); 12 UO di Ematologia, Ospedale Sant’Eugenio, 00144 Roma, Italy; pniscola@gmail.com; 13 Dipartimento di Oncoematologia, AOU San Giovanni di Dio e Ruggi D’Aragona, 84125 Salerno, Italy; biaserio@gmail.com; 14 Department of Precision and Translational Medicine with Ionian Area, “Aldo Moro” University School of Medicine, 70121 Bari, Italy; rino.musto56@gmail.com; 15 Unit of Hematology and Stem Cell Transplantation, AOUC Policlinico, 70124 Bari, Italy; 16 UO di Ematologia, Ospedale L’Annunziata, 87100 Cosenza, Italy; ernesto.vigna@aocs.it; 17 UOC Ematologia e Trapianto di Cellule Staminali Emopoietiche, Policlinico Foggia Ospedaliero-Universitario, 71122 Foggia, Italy; lmelillo@ospedaliriunitifoggia.it; 18 Nephology, Dialysis and Transplantation Unit-GOM “Bianchi-Melacrino-Morelli”, 89124 Reggio Calabria, Italy; rtripepi@ifc.cnr.it; 19 Department of Hematology/Oncology, The Institute of Medical Science, The University of Tokyo, Tokyo 108-0071, Japan; yasuhito.nannya@gmail.com; 20 Institute for the Advanced Study of Human Biology, Kyoto University, Kyoto 606-8303, Japan; sogawa-tky@umin.ac.jp

**Keywords:** acute myeloid leukemia, azacitidine, disease-free survival, malignancy-related genes

## Abstract

The achievement of complete remission (CR) is crucial for acute myeloid leukemia (AML) patients undertaking curative therapy, but relapse often occurs within months, highlighting the need for strategies to prolong disease-free survival (DFS). Our phase III study compared the efficacy and safety of azacitidine (AZA) to best supportive care (BSC) in elderly AML patients who achieved CR following intensive induction and consolidation therapy. This ancillary study (QOL-ONE Trans-2) evaluated biological changes in bone marrow using Next-Generation Sequencing (NGS). We analyzed baseline, randomization, and 6-month post-remission samples from 24 patients (median age of 71 and 12 males). High-throughput NGS targeted 350 myeloid malignancy-related genes, considering variants with a variant allele frequency ≥ 4%. At diagnosis, all patients had 5 to 17 (median = 10) mutations, with DNMT3A (42%), NPM1 (33%), and TET2 (33%) being most frequent. FANCA mutations in four patients were linked to a higher relapse risk (HR = 4.96, *p* = 0.02) for DFS at both 2 and 5 years. Further HLA-specific NGS analyses are ongoing to confirm these results and their therapeutic implications.

## 1. Introduction

Achieving complete remission (CR) is a critical benchmark for patients with acute myeloid leukemia (AML) receiving curative-intent therapy [1]. Factors such as age, comorbidities, and biological characteristics of leukemia in older adults [2] influence their ability to tolerate therapy, often leading to poorer outcomes and lower CR rates compared to younger patients [3,4]. Independent of their age, most patients who attain remission through induction therapy for AML will relapse within months, unless further therapy is administered [5]. As a result, there has been sustained interest in the use of maintenance therapies with reduced intensity following completion of the intensive treatment phase, aiming to prolong remission, increase survival and increase the likelihood of cure [6,7,8].

Azacitidine (AZA), a hypomethylating agent with established antileukemic properties, is widely used alone or in combination with other drugs for the frontline treatment of AML patients deemed unfit for intensive chemotherapy due to its favorable safety profile [9,10,11,12,13].

A recent study evaluated the use of AZA as a 1-year maintenance therapy following CR in elderly AML patients, demonstrating improved relapse-free survival (RFS), although no significant impact on overall survival (OS) was observed [14]. Furthermore, a randomized placebo-controlled clinical trial evaluating oral AZA formulation (CC-486) as a maintenance therapy in patients aged 55 years and older after induction treatment showed significant improvements in both OS and RFS in those receiving the experimental drug.

The phase III “QoLESS AZA-AMLE” randomized trial aimed to assess the efficacy of long-term AZA maintenance compared to placebo in elderly AML patients who achieved first CR after a homogeneous intensive induction and consolidation phase [15]. The trial evaluated the efficacy of subcutaneous AZA post-remission treatment to best supportive care (BSC) in elderly AML patients, with the primary endpoint being the difference in disease-free survival (DFS) from CR to relapse or death. Patients aged 61 years or older with newly diagnosed AML received two courses of induction chemotherapy (daunorubicin and cytarabine) followed by cytarabine consolidation. After achieving CR, 54 patients were randomized in a 1:1 ratio to receive either BSC or AZA. After 2 years, the median DFS was 6.0 months for BSC recipients compared to 10.8 months for AZA recipients. After 5 years, median DFS was 6.0 months in the BSC arm compared to 10.8 months in the AZA arm. Significant benefit was observed by AZA on DFS at 2 and 5 years in patients aged >68 years (HR = 0.34, 95% CI: 0.13–0.90, *p* = 0.030 and HR = 0.37, 95% CI: 0.15–0.93, *p* = 0.034, respectively). No deaths occurred before relapse and neutropenia was the most frequent adverse event. Patient-reported outcome measures did not differ between the two study arms. In conclusion, AZA post-remission therapy is feasible, safe, and favorable, particularly in AML patients aged >68 years.

We report on the ancillary “Translational study, research on Azacitidine Post-Remission Therapy of Acute Myeloid Leukemia in Elderly Patients (QOL-ONE Trans-2)”, to evaluate biological changes through Next-Generation Sequencing (NGS) using a custom genomic panel, courtesy of Prof. Seishi Ogawa (University of Kyoto, Kyoto, Japan), for all subjects with available vials.

## 2. Results

### 2.1. Description of the Study Cohort

In this manuscript, we present NGS data in randomized patients performed at disease diagnosis (baseline visit), at CR post-induction chemotherapy (randomization visit), and at 6 months post-randomization.

Bone marrow samples were available for 63 patients at baseline; however, due to time-related preservation defects, biological samples for 10 patients were not evaluable.

Baseline characteristics of the 53 evaluable patients are presented in Table 1.

All 53 patients presented mutations at diagnosis with a range of 3 to 19 (median = 10) simultaneous mutations, the most frequent being DNMT3A (38%), TET2 (28%), NPM1 (23%), and DST (23%). After induction chemotherapy, 29 patients did not achieve CR, therefore the bone marrow samples of post-induction therapy phase were not collected. The sample data of the 24 patients who had reached CR and were randomized in 5-AZA arm (11 patients) or in the BSC arm (13 patients) are here reported. In Table 2, baseline characteristics of the 24 patients in CR are shown.

**Figure 1:**
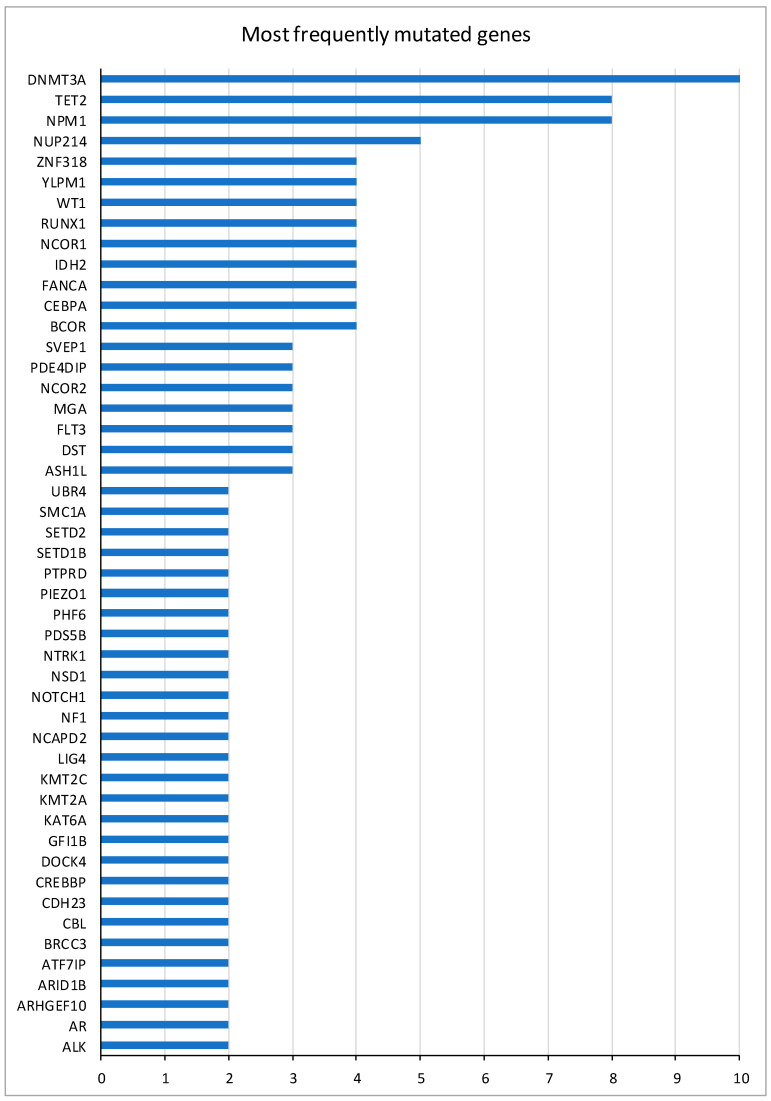
Most frequently mutated genes of patients at diagnosis.

All 24 patients presented mutations at diagnosis with a range of 5 to 17 (median = 10) simultaneous mutations, the most frequent being DNMT3A (42%), NPM1 (33%), and TET2 (33%). In Figure 1, the most frequently mutated genes at diagnosis are represented.

The following gene mutations occurred only once: ABL1, ACIN1, ADA2, ALAS2, ANKRD26, ARID1A, ARID2, ASXL1, ATG2B, BCORL1, CALR, CBFA2T3, CBLB, CDC25C, CDKN2B, CHM, CTCF, DAZAP1, DCAF8L1, DIS3, DYNC2H1, ELANE, FANCD2, FANCG, FANCM, FBXW7, FMC1, FMC1_LUC7L2, GIGYF2, HCFC1, HCN1, HSPA9, IDH1, JAK1, KDM5A, KDM6A, KLF1, KMT2D, KRAS, LIN28A, MBD4, MBNL1, MDM2, MET, MYC, MZF1, NFE2, NIPBL, NOL3, NTRK3, NXF1, PALB2, PARN, PDGFRA, PDGFRB, PIK3CG, PRF1, PRPF8, PTPN11, PUS1, PXDNL, ROBO1, ROS1, RTEL1, RUNX1T1, SAMD9, SF1, SF3A1, SLIT3, SMC3, SNX13, SRCAP, SRP54, SRP72, SRSF2, STAG1, SYK, TLR2, TNRC18, VEGFA, ZBTB7A, ZEB2, and ZFPM1.

### 2.2. Primary Endpoints

#### 2.2.1. Effect Modifications by Gene Mutations at AML Diagnosis

The effect modification by gene mutations at AML diagnosis on the relationship between AZA versus BSC and relapse were evaluated at 2 and 5 years.

In Table 3 and Table 4, hazard ratios (HR) and 95% confidence intervals (CI) of relapse at 2 and 5 years of most frequently occurring mutations are shown.

#### 2.2.2. Effect Modifications by Gene Mutations at Randomization

**Figure 2:**
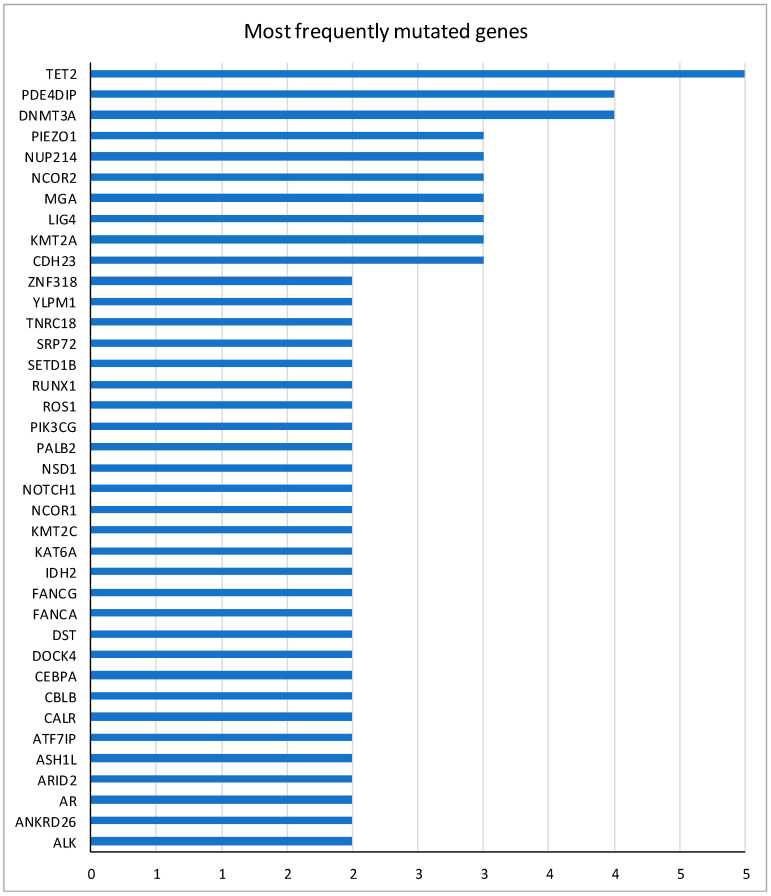
Most frequently mutated genes at randomization.

In Figure 2, the most frequent mutated genes at randomization are represented. Two out of four cases lost the FANCA gene mutation at randomization. No other effect modification by gene mutations at random (MRD) on the relationship between AZA versus BSC and relapse in elderly AML patients receiving induction chemotherapy followed by post-remission BSC versus AZA maintenance was observed at 2 and 5 years (*p* = 0.14 to *p* = 0.98).

### 2.3. Secondary Endpoints

#### DFS in All Randomized Patients and Stratified by Mutated FANCA Gene at Diagnosis

**Figure 3:**
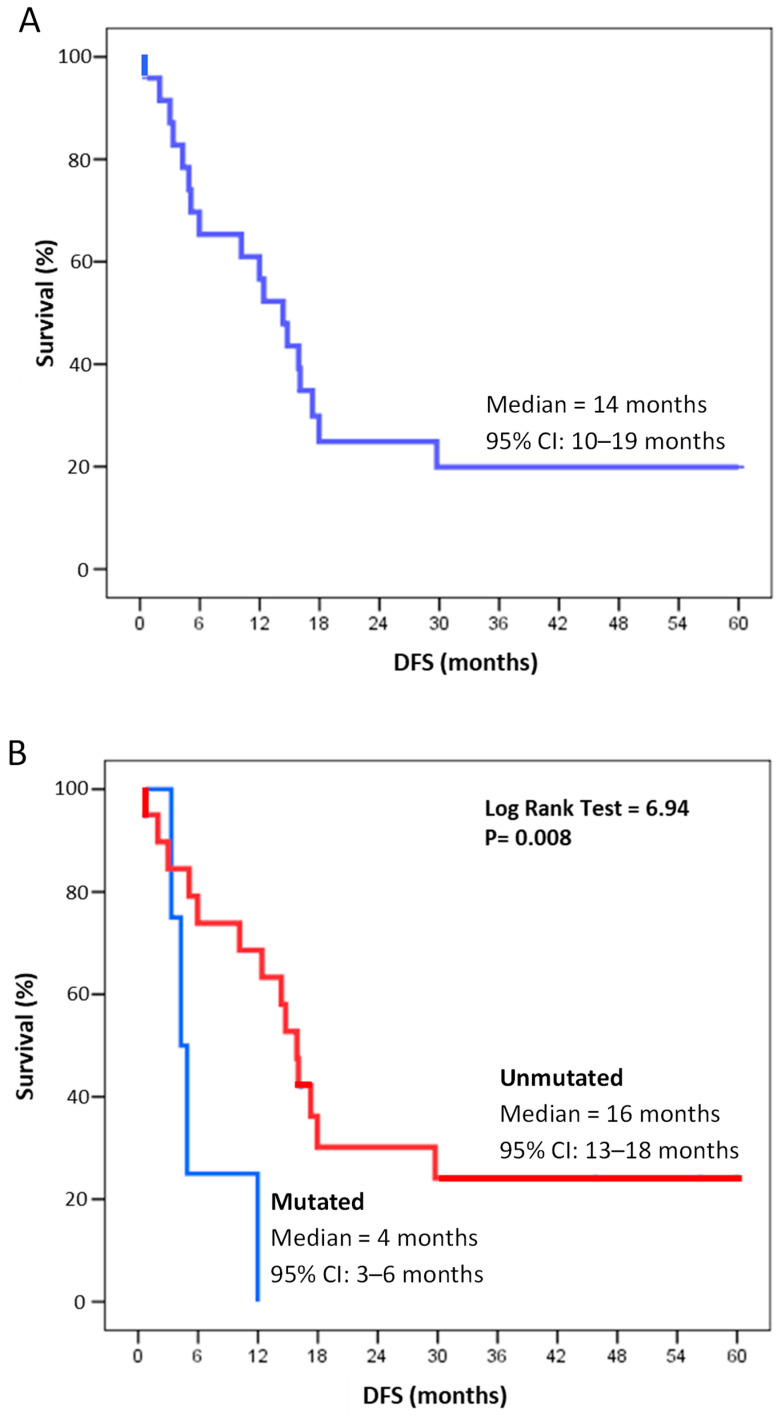
Disease free survival up to 5 years in all randomized patients (**A**) and those stratified by unmutated (N = 20) and mutated (N = 4) FANCA genes (**B**) at diagnosis. DFS = disease-free survival.

Kaplan–Meier survival curves for all 24 randomized patients (Figure 3A) and patients stratified for the presence of unmutated (N = 20) and mutated (N = 4) FANCA genes (Figure 3B) are shown. Median DFS in randomized patients was 14 months (95% CI: 10–19 months), higher in patients with unmutated FANCA genes (median DFS of 16 months, 95% CI: 13–18) versus those with mutated FANCA genes (median DFS of 4 months, 95% CI: 3–6 months, Log Rank test, *p* = 0.008) (Figure 3B).

A case summary of the four patients with the mutated FANCA gene is shown in Table 5.

## 3. Discussion

AML relapse poses a significant challenge, with low survival rates despite various interventions [16,17].

In the present analysis, samples from 24 patients revealed 5–17 mutations each (median = 10), with DNMT3A, NPM1, and TET2 being most frequent. Only FANCA (mutated in four patients) significantly correlated with higher relapse risk (HR = 4.96, *p* = 0.02).

Several other genes showed a similar/higher rate of mutation and, though associated with DFS, they did not achieve statistical significance likely due to the limited sample size. Indeed, mutations in genes such as ZNF318, WT1, TET2, and DNMT3A are recognized to significantly influence the prognosis of AML [18]. ZNF318 mutations have been linked to altered gene regulation, potentially affecting leukemia progression and patient outcome. WT1 and TET2 mutations have also been shown to occur in about 6–15% of AML cases, often predicting poor prognosis due to their association with higher relapse rates [19,20,21]. DNMT3A mutations, one of the most frequent in AML, found in 20–30% of cases, are associated with adverse outcomes [22].

The current study highlights the prognostic significance of specific gene mutations, particularly FANCA, in predicting DFS and relapse in elderly patients who achieved CR after intensive induction and consolidation therapy.

Mutations in the FANCA gene were associated with a significantly increased HR of relapse, indicating a poorer prognosis for patients with these mutations. This finding aligns with previous studies that have demonstrated the involvement of FANCA and other Fanconi anemia (FA) genes in hematologic malignancies. In addition, emerging lines of evidence indicate that the FA pathway constitutes a general surveillance mechanism for the genome by protecting against a variety of DNA replication stresses [23]. Consequently, studies have been undertaken to improve our understanding of DNA repair signaling that is regulated by the FA pathway, and the potential role of DNA lesions underlying the FA pathophysiology for the treatment of FA and FA-associated cancers.

Pawlikowska and colleagues demonstrated that loss of the Fanconi anemia pathway, known to control genetic instability, promotes the expansion of leukemic cells carrying oncogenic mutations rather than mutation formation [24]. Furthermore, in a study by D’Andrea and colleagues the role of the FA pathway in DNA repair mechanisms was emphasized, suggesting that mutations in FANCA could impair the repair of DNA crosslinks, thereby contributing to leukemogenesis [25]. Furthermore, Voso et al. observed that FA gene mutations, including FANCA, were prevalent in therapy-related myeloid neoplasms, highlighting their potential role in the pathogenesis of secondary AML [26].

The association of FANCA mutations with poor prognosis in AML has also been corroborated by other studies. Zhang et al. identified FANCA mutations as a common event in AML patients and linked these mutations with adverse outcomes, particularly in terms of DFS and overall survival (OS). Similarly, Tischkowitz et al. (2015) found that germline mutations in FANCA were associated with increased AML risk, further highlighting the gene’s relevance in the disease’s etiology and progression. However, in a separate study by Chang et al. compared to the FA wild-type group, a decrease in the expression of FNACD2, FANCI, and RAD51C was observed in the FA mutation group. Interestingly, the FA mutation group exhibited a more favorable clinical overall survival prognosis [27].

However, the frequency of FANCA mutations and their prognostic impact can vary across studies. In the current study, FANCA mutations were present in 4 out of 24 patients (approximately 17%), which is consistent with some reports but higher than others. This suggests potential variability due to differences in patient demographics or study methodologies. Reinforcing this idea, Steinberg-Shemer performed a characterization and genotype-phenotype correlation of patients with FA in a multi-ethnic population. Patients with *FANCA* mutations developed cancer at a significantly older age compared to patients with mutations in other Fanconi genes; however, overall survival was not found to be dependent on the causative gene [28].

A phase II trial explored AZA in 39 patients with persistent disease or early relapse post-HCT, finding a 30% response rate, including three CR [29]. The most commonly mutated genes at the time of relapse included TP53 (48%), TET2 (33%), and DNMT3A (14%). Mutations in TP53 were significantly associated with poor responsiveness to AZA (OR 3.08, 95% CI: 1.1–9.0; p = 0.04] and inferior survival; HR = 3.04, 95% CI: 1.3–5.8; *p* = 0.02]. We also observed a high number of mutations for the TET2 gene (10 mutations); however, the risk of 2- and 5-year DFS was not statistically significant (*p* = 0.73 and *p* = 0.52, respectively).

Our findings on FANCA mutations suggest potential markers for tailoring post-remission therapy to prolong DFS.

Overall, these studies contribute valuable insights into the genetic factors influencing treatment outcomes in AML/MDS, advocating for personalized approaches based on mutational profiles to enhance therapeutic strategies and improve patient prognosis.

The differential impact of FANCA mutations on AML prognosis highlights the importance of personalized medicine in this field. Patients with FANCA mutations may benefit from more aggressive monitoring and potentially alternative therapeutic strategies to mitigate the higher risk of relapse.

Further research is warranted to validate these findings and explore their underlying mechanisms. Specifically, additional studies should investigate the functional consequences of FANCA mutations in AML cells and their interactions with the bone marrow microenvironment. The use of larger patient cohorts and diverse populations will also be essential to generalize these results.

### 3.1. Study Limitations

Our analysis was based on a small number of patients (24 patients were included) with wide heterogeneity of mutations evaluated. A wide range of 5 to 17 simultaneous mutations per patient may potentially complicate the analysis and interpretation of the specific impact of a given gene. Data were collected at baseline, randomization, and 6 months post-remission, potentially missing long-term effects. Results may not be applicable to younger AML patients due to the specific age group studied (median age 71). These limitations suggest that while the findings are promising, they should be interpreted cautiously and validated in larger, more diverse cohorts.

### 3.2. Conclusion

The present study adds valuable insights into the prognostic significance of FANCA mutations in elderly AML patients. The association of FANCA mutations with increased relapse risk with improved DFS highlights the potential for these genetic markers to inform treatment decisions and risk stratification. Future research should aim to elucidate the mechanisms underlying these associations and explore their implications for personalized AML therapy.

## 4. Materials and Methods

### 4.1. Study Design and Patient Population

The study was performed on available biological samples collected at baseline, randomization, and 6-month post-remission. For study design and patient population refer to the published report [15]. 

### 4.2. Inclusion and Exclusion Criteria

Samples of patients that have been enrolled in the Azacitidine Post-Remission Therapy for Elderly Patients with AML: A Randomized Phase-3 Trial [15] are included if they have a baseline (at diagnosis) bone marrow sample available. The lack of an available baseline bone marrow sample is an exclusion criterion for the present study.

### 4.3. Study Endpoints

The primary endpoints of the study were to evaluate how gene mutations at AML diagnosis modify the effect of AZA versus BSC on relapse, and how gene mutations at randomization (minimal residual disease, MRD) modify the effect of AZA versus BSC on relapse in elderly AML patients who received induction chemotherapy followed by either post-remission BSC or AZA maintenance.

### 4.4. Study Procedures

Next-Generation Sequencing (NGS) assessment was performed on bone marrow samples from patients enrolled in the study by the Medical Genetics Unit, Grande Ospedale Metropolitano Bianchi Melacrino Morelli, Reggio Calabria, Italy. Samples were serially collected at different time-points as required by the protocol. The genomic DNA was extracted from bone marrow (preserved in DMSO/Trizol or pellet) with a Trizol/chloroform method or QIAmp DNA Mini Kit (QIAamp DNA Mini and Blood Mini Handbook: www.qiagen.com/HB-0329) following the manufacturer’s instructions and stored at −80 °C until the time of use.

DNA was subjected to high throughput NGS using genes commonly mutated in myeloid malignancies and prepared with a home-set of genes for Illumina (see Supplementary Materials, courtesy of Prof. Seishi Ogawa, Kyoto, Japan).

Targeted sequencing was performed using a custom DNA bait library (Sure Select; Agilent Technology) as previously described [30].

Sequencing libraries were generated according to an Illumina paired-end library protocol. The targets were subjected to massive sequencing using Hiseq 2000 (Illumina), with sufficient read coverage. Only variants with high-quality reads were considered. Variants were annotated using Agilent Technologies Alissa Interpret v5.4.2, the Platform data set was 44_1, RefSeq Transcripts v205, Genome build GRCh37.p13, and the database of functional predictions for non-synonymous SNPs was dbNSFP, dbSNP build 151, and NCBI ClinVar 2022-12.

A Variant Allele Frequency (VAF) ≥ 4% was considered an appropriate threshold for minimal burden of clonality to be reported.

### 4.5. Statistical Analysis

Data were summarized as mean and standard deviation, median, and interquartile range, or absolute frequency and percentage, as appropriate.

The effect modification by gene mutations at AML diagnosis and at randomization on the relationship between AZA versus BSC and relapse was investigated by Cox proportional hazard model and by applying the standard linear combination method. In Cox models, data were expressed as hazard ratios (HRs), 95% confidence intervals, and *p*-values. In these models, potential confounders were taken into account. The relationship between gene mutations at diagnosis and DFS at 2 and 5 years for all patients, independently of allocation arm, was evaluated by Kaplan–Meier analysis and univariate COX regression. All calculations were performed using the SPSS version 13 for Windows software.

## Figures and Tables

**Table 1:** Baseline patient characteristics.

Characteristics	N = 53
Age, median years (IQR)	71 (66–74)
Male, n (%)	28 (52.8)
AML de novo, n (%)	46 (86.8)
Hemoglobin (Hb), mean g/dL (±SD)	9.4 ± 1.6
White blood cell (WBC) × 10^3^, median (IQR)	15.6 (2.8–37.8)
Platelet (PLT) × 10^3^, median (IQR)	61.5 (27.0–85.0)
WHO classification, n (%)	
*AML with minimal differentiation*	8 (15.1)
*Acute myelomonocytic leukemia*	9 (17.0)
*AML with myelodysplasia-related changes*	9 (17.0)
*AML with maturation*	12 (22.6)
*Acute monoblastic and monocytic leukemia*	5 (9.4)
*AML without maturation*	5 (9.4)
*AML with recurrent genetic abnormalities*	3 (5.7)
*Therapy-related myeloid neoplasms*	1 (1.9)
*Acute erythroid leukemia*	1 (1.9)
Cytogenetic risk profile, n (%)	
*Intermediate*	40 (75.5)
*Poor*	7 (13.2)
*Not evaluable*	6 (12.3)

AML = acute myeloid leukemia, WHO = World Health Organization.

**Table 2:** Baseline characteristics of randomized CR patients.

Characteristics	AZA (*n* = 11)	BSC (*n* = 13)	All Patients (*n* = 24)
Age, median years (IQR)	70 (66–75)	73 (65–74)	71 (65–74)
Male, n (%)	6 (54.5)	6 (46)	12 (50)
AML de novo, n (%)	9 (82)	13 (100.0)	22 (92)
Hemoglobin (Hb), mean g/dL (±SD)	9.3 ± 0.9	9.4 ± 1.4	9.4 ± 1.2
White blood cell (WBC) × 10^3^, median (IQR)	3.1 (1.7–40.2)	17.1 (2.7–25.1)	15.6 (1.8–28.9)
Platelet (PLT) × 10^3^, median (IQR)	43 (26–63)	29 (22–71)	41 (24–65)
WHO classification, n (%)			
*AML with minimal differentiation*	1 (9.1)	2 (15.4)	3 (12.5)
*Acute myelomonocytic leukemia*	3 (27.3)	2 (15.4)	5 (20.8)
*AML with myelodysplasia-related changes*	2 (18.2)	1 (7.7)	3 (12.5)
*AML with maturation*	2 (18.2)	3 (23.1)	5 (20.8)
*Acute monoblastic and monocytic leukemia*	1 (9.1)	3 (23.1)	4 (16.6)
*AML without maturation*	1 (9.1)	-	1 (4.2)
*AML with recurrent genetic abnormalities*	-	1 (7.7)	1 (4.2)
*Therapy-related myeloid neoplasms*	-	1 (7.7)	1 (4.2)
*Acute erythroid leukemia*	1 (9.1)	-	1 (4.2)
Cytogenetic risk profile, n (%)			
*Intermediate*	8 (72.7)	11(84.6)	19 (79.2)
*Poor*	1 (9.1)	2 (15.4)	3 (12.5)
*Not evaluable*	2 (18.2)	-	2 (8.3)

AML = acute myeloid leukemia, AZA = azacitidine, BSC = best supportive care, WHO = World Health Organization.

**Table 3:** DFS at 2 years.

Gene	Patients with Mutated Genes (N)	Patients with Unmutated Genes (N)	HR (95% CI)	*p*-Value
DNMT3A	10	14	0.45 (0.15–1.30)	0.14
TET2	8	16	1.20 (0.44–3.27)	0.73
NPM1	8	16	0.47 (0.15–1.48)	0.17
NUP214	5	19	0.72 (0.23–2.23)	0.56
ZNF318	4	20	1.92 (0.62–5.95)	0.28
YLPM1	4	20	0.37 (0.85–1.65)	0.19
WT1	4	20	1.39 (0.39–4.88)	0.61
RUNX1	4	20	0.34 (0.15–2.85)	0.57
NCOR1	4	20	0.34 (0.08–1.50)	0.15
IDH2	4	20	0.52 (0.12–2.31)	0.39
FANCA	4	20	4.96 (1.34–18.35)	**0.02**
CEBPA	4	20	1.01 (0.29–3.53)	0.98
BCOR	4	20	1.29 (0.29–5.74)	0.74

CI = confidence interval, DFS = disease-free survival, HR = hazard ratio. Statistically significant *p* values are represented by bold text.

**Table 4:** DFS at 5 years.

Gene	Patients with Mutated Genes (N)	Patients with Unmutated Genes (N)	HR (95% CI)	*p*-Value
DNMT3A	10	14	0.53 (0.19–1.43)	0.21
TET2	8	16	1.38 (0.53–3.57)	0.52
NPM1	8	16	0.53 (0.19–1.62)	0.28
NUP214	5	19	0.69 (0.22–2.11)	0.51
ZNF318	4	20	1.92 (0.62–5.95)	0.26
YLPM1	4	20	0.58 (0.16–2.01)	0.39
WT1	4	20	1.26 (0.36–4.38)	0.72
RUNX1	4	20	0.60 (0.14–2.63)	0.50
NCOR1	4	20	0.49 (0.14–1.74)	0.27
IDH2	4	20	0.49 (0.11–2.15)	0.34
FANCA	4	20	4.96 (1.34–18.35)	**0.02**
CEBPA	4	20	0.93 (0.27–3.24)	0.91
BCOR	4	20	0.74 (0.29–5.74)	0.74

CI = confidence interval, DFS = disease-free survival, HR = hazard ratio. Statistically significant *p* values are represented by bold text.

**Table 5:** Patients with FANCA-mutated gene at diagnosis.

Case Number	Arm	FANCA at Randomization	DFS (Months)
Patient #1	AZA	Unmutated	3
Patient #2	BSC	Unmutated	12
Patient #3	BSC	Mutated	5
Patient #4	BSC	Mutated	4

AZA = azacitidine, BSC = best supportive care, DFS = disease-free survival.

## Data Availability

Supplementary Material accompanies this paper. Raw data are available upon request from the corresponding author (E.N.O.).

## Supplementary Materials

The following supporting information can be downloaded at: https://www.mdpi.com/article/10.3390/ijms252111646/s1.
