# Supplementary material for: Translational Research on Azacitidine Post-Remission Therapy of Acute Myeloid Leukemia in Elderly Patients (QOL-ONE Trans-2)
Source: Int J Mol Sci. 2024 Oct 30;25(21):11646. doi: 10.3390/ijms252111646 (PMC11545844; doi:10.3390/ijms252111646)
Supplement: Supplementary file 1 [file ijms-25-11646-s001.zip › ijms-3254905-supplementary.pdf]

Home-set of genes for Illumina
Browser position chr1:1718703-1718942
track name="Covered" description="Agilent SureSelect DNA - myeloid_1905 - Genomic regions overlapped by probes" db=hg19

chr1 MTOR
chr1 CLCN6
chr1 LIN28A
chr1 ARID1A
chr1 MPL
chr1 JAK1
chr1 GFI1
chr1 NRAS
chr1 NOTCH2
chr1 PDE4DIP
chr1 HAX1
chr1 RIT1
chr2 E2F6
chr2 DNMT3A
chr2 ASXL2
chr2 ALK
chr2 FANCL
chr2 SF3B1
chr2 IDH1
chr3 TRNT1
chr3 FANCD2
chr3 VHL
chr3 RAF1
chr3 NBEAL2
chr3 SETD2
chr3 GATA2
chr3 MBD4
chr3 ATR
chr3 GPR171
chr3 MBNL1
chr3 MECOM
chr3 PIK3CA
chr3 RPL35A
chr4 FGFR3
chr4 DHX15
chr4 PDGFRA

chr4 KIT
chr4 KDR
chr4 SRP72
chr4 TET2
chr4 FBXW7
chr4 TLR2
chr4 IRF2
chr5 SDHA
chr5 TERT
chr5 IRX1
chr5 C1QTNF3
chr5 DNAJC21
chr5 NIPBL
chr5 HCN1
chr5 APC
chr5 IRF1
chr5 CDC25C
chr5 HSPA9
chr5 PCDHA1
chr5 CSNK1A1
chr5 CSF1R
chr5 PDGFRB
chr5 NPM1
chr5 FGFR4
chr5 NSD1
chr5 DDX41
chr5 NHP2
chr6 HIST1H3F
chr6 ZNF318
chr6 VEGFA
chr6 PHIP
chr6 ROS
chr6 MYB
chr6 ECT2L
chr6 ARID1B
chr7 TNRC18
chr7 SNX13
chr7 IKZF1
chr7 EGFR
chr7 SBDS
chr7 CDK6

chr7 SAMD9
chr7 SAMD9L
chr7 TAF6
chr7 STAG3
chr7 GNB2
chr7 GIGYF1
chr7 CUX1
chr7 KMT2E
chr7 PIK3CG
chr7 DOCK4
chr7 MET
chr7 SMO
chr7 LUC7L2
chr7 BRAF
chr7 EZH2
chr7 SMARCD3
chr7 KMT2C
chr7 XRCC2
chr8 ARHGEF10
chr8 ESCO2
chr8 FGFR1
chr8 KAT6A
chr8 PXDNL
chr8 TERF1
chr8 RUNX1T1
chr8 BAALC
chr8 RAD21
chr8 MYC
chr9 JAK2
chr9 PTPRD
chr9 CDKN2A
chr9 GNE
chr9 GNAQ
chr9 HNRNPK
chr9 NTRK2
chr9 SYK
chr9 ERCC6L2
chr9 SVEP1
chr9 ABL1
chr9 NUP214
chr9 SETX

chr9 TSC1
chr9 GFI1B
chr9 NOTCH1
chr10 DCLRE1C
chr10 MLLT10
chr10 ANKRD26
chr10 ITGB1
chr10 RET
chr10 PRF1
chr10 CDH23
chr10 WAPAL
chr10 PTEN
chr10 NOLC1
chr10 SMC3
chr10 FGFR2
chr11 HRAS
chr11 NUP98
chr11 TPP1
chr11 SBF2
chr11 RRAS2
chr11 PIK3C2A
chr11 WT1
chr11 SPI1
chr11 NXF1
chr11 SF1
chr11 MAP4K2
chr11 KLC2
chr11 CCND1
chr11 PAK1
chr11 PICALM
chr11 EED
chr11 MRE11A
chr11 DYNC2H1
chr11 ATM
chr11 KMT2A
chr11 CBL
chr11 ETS1
chr12 KDM5A
chr12 CCND2
chr12 NCAPD2
chr12 ETV6

chr12 CDKN1B
chr12 ATF7IP
chr12 ETNK1
chr12 KRAS
chr12 YARS2
chr12 ARID2
chr12 KMT2D
chr12 NFE2
chr12 RPS26
chr12 ERBB3
chr12 CDK4
chr12 MDM2
chr12 SH2B3
chr12 PTPN11
chr12 SETD1B
chr12 NCOR2
chr12 PUS1
chr13 FLT3
chr13 BRCA2
chr13 BRCA2
chr13 PDS5B
chr13 ELF1
chr13 RB1
chr13 DIS3
chr14 ACIN1
chr14 TINF2
chr14 SRP54
chr14 FANCM
chr14 RPS29
chr14 YLPM1
chr14 DICER1
chr14 GLRX5
chr14 ATG2B
chr14 GSKIP
chr14 BCL11B
chr14 AKT1
chr15 NOP10
chr15 RAD51
chr15 MGA
chr15 CDAN1
chr15 C15ORF65

chr15 MAP2K1
chr15 IDH3A
chr15 825EFTUD1
chr15 RPS17
chr15 NTRK3
chr15 FANCI
chr15 IDH2
chr15 BLM
chr15 IGF1R
chr16 TSC2
chr16 CREBBP
chr16 PARN
chr16 MYH11
chr16 RPS15A
chr16 ACSM2A
chr16 PALB2
chr16 RBBP6
chr16 SRCAP
chr16 SETD1A
chr16 CBFB
chr16 NOL3
chr16 CTCF
chr16 CDH1
chr16 TERF2
chr16 RFWD3
chr16 GSE1
chr16 ZFPM1
chr16 PIEZO1
chr16 CBFA2T3
chr16 FANCA
chr17 PRPF8
chr17 TP53
chr17 WRAP53
chr17 CTC1
chr17 RPL26
chr17 NCOR1
chr17 NF1
chr17 SUZ12
chr17 MED1
chr17 ERBB2
chr17 IKZF3

chr17 STAT5B
chr17 STAT3
chr17 RPL27
chr17 BRCA1
chr17 G6PC3
chr17 UBTF
chr17 KANSL1
chr17 RAD51C
chr17 PPM1D
chr17 BRIP1
chr17 DCAF7
chr17 SRSF2
chr18 SETBP1
chr18 DCC
chr18 TCF4
chr18 BCL2
chr19 ELANE
chr19 STK11
chr19 DAZAP1
chr19 DOT1L
chr19 GNA11
chr19 ZBTB7A
chr19 RPS28
chr19 DNM2
chr19 SMARCA4
chr19 EPOR
chr19 KLF1
chr19 CALR
chr19 JAK3
chr19 CEBPA
chr19 AKT2
chr19 RPS19
chr19 ERF
chr19 RRAS
chr19 PRMT1
chr19 U2AF2
chr19 MZF1
chr20 RAD21L1
chr20 IDH3B
chr20 BCL2L1
chr20 ASXL1

chr20 PIGT
chr20 PTPN1
chr20 BCAS1
chr20 AURKA
chr20 GNAS
chr20 RTEL1
chr21 NRIP1
chr21 LTN1
chr21 RUNX1
chr21 ERG
chr21 ETS2
chr21 U2AF1
chr22 CECR1
chr22 MN1
chr22 CHEK2
chr22 NF2
chr22 SF3A1
chr22 CSF2RB
chr22 EP300
chrX CSF2RA
chrX FANCB
chrX ZRSR2
chrX BCOR
chrX USP9X
chrX KDM6A
chrX NDUFB11
chrX GATA1
chrX PIM2
chrX SMC1A
chrX TSR2
chrX ALAS2
chrX AR
chrX MED12
chrX HDAC8
chrX ABCB7
chrX ATRX
chrX UBE2A
chrX STAG2
chrX BCORL1
chrX PHF6
chrX HCFC1

chrX DKC1
chrX BRCC3
chrY CSF2RA
